# Mind, Body and Boundaries: Self-Presentation on the Nordic LGBTQ Online Dating Scene

**DOI:** 10.3389/fpsyg.2019.02770

**Published:** 2019-12-10

**Authors:** Emelie Louise Miller

**Affiliations:** Department of Psychology and Social Work, Mid Sweden University, Östersund, Sweden

**Keywords:** sexuality, gender, online dating, self-presentation, Sweden, LGBTQ, minorities

## Abstract

Online dating is continually on the rise and nowadays a widely used and accepted way to find different kinds of companionship. This relatively new interpersonal phenomenon has provided an especially important virtual space for non-heterosexuals. Previous research on behaviors and trends on dating communities online for sexual minorities has focused primarily on sites for gay men in Anglo-Saxon countries. The purpose of the present study is to examine self-presentations on the Nordic LGBTQ online dating scene and possible gender-dependent differences in self-presentation. The Nordic countries are commonly perceived as progressive in issues regarding gender equality and LGBTQ rights. The countries on average also have low population density with large rural areas and consequently limited scenes for non-heterosexuals. A testimony of this is the study’s selected dating site, which is based in Sweden but encompasses the neighboring countries and markets itself as a Nordic meeting venue. The present study embarks on new territory within psychology-, gender-, and queer research by examining self-presentations on a mixed-gender LGBTQ dating site, situated in the supposedly liberal Nordic countries. Based on qualitative and quantitative data from a stratified sample of 716 cis-gendered, predominantly Swedish online dating profiles, on a well-established Nordic online dating site for non-heterosexual men and women, statistical calculations and a thematic analysis (TA) were executed. The findings show that central self-presentations concern mind versus body, lust and longings, and boundaries, where gender frequently functions as the dividing line. Women self-present more through personality and romantic longings compared to men, who to a higher degree emphasizes body, and lust. Self-presentation is also expressed through resistance against boundary-breaking contact on the site. The boundaries that are guarded regard age, anti-racism and most pronounced – boundaries against male harassment of non-heterosexual women. The implications of self-presentation, possible discrimination and misrepresentation on the Nordic LGBTQ online dating scene are discussed.

## Introduction

During the last couple of decades the Internet has become a central venue for connecting with people. Everyone with Internet access can nowadays form various relationships with perfect strangers, no longer restricted to social contacts gained through family, friends, school and workplaces (Rosenfeld and Reuben, 2012). Online dating sites and apps have transformed the dating landscape and increasingly compete with, and overshadow, conventional spaces for singles (Finkel et al., 2012; Nash, 2013). Many members of online dating sites emphasize the possibility to pursue multiple potential partners simultaneously in an endless sea of profiles (Jones, 2005; Hobbs et al., 2017). This shopping mentality has however, not rendered longing for romance and love obsolete (Rosenfeld and Byung-Soo, 2005). Attitudes toward online dating have grown progressively positive as more and more people use such services (Smith and Anderson, 2015). The freedom to choose one’s sexual and romantic partners is particularly crucial for non-heterosexual men and women, who historically and continually face difficulties and marginalization owing to their sexuality (Brown et al., 2005; Shield, 2014; Callander et al., 2015). Since non-heterosexuals are especially likely to meet online (Rosenfeld and Reuben, 2012), dating sites are meaningful to explore as intersections of e.g., sexuality, gender, and ethnicity increasingly are co-constructed and defined by such internet-based technology (Murray and Ankerson, 2016). This sort of co-construction is key in understanding if and in what way people are represented and how they self-present. In the Nordic context, here exemplified by the Swedish conditions, the public opinion is in general supportive of sexual minorities (Peterson et al., 2018). To a varying degree, all political parties in the Swedish parliament embrace LGBTQ rights, as does the Swedish military (Sundevall and Persson, 2016; Svensson, 2016; Carlson-Rainer, 2017). An increasing numbers of health and social care providers are educated and LGBTQ-certified by the main Swedish LGBTQ organization RFSL (Kottorp et al., 2016). Heteronormativity is however, still strong and well in Sweden and the social control continues to question non-heterosexuals normality (Magnusson, 2011; Timofejevs-Henriksson, 2011). Public displays of non-heterosexuality, in Swedish Pride parades, over-represent young, highly educated and politically left oriented people (Peterson et al., 2018). Mental health problems and suicide risks are more pronounced among Swedish non-heterosexuals compared to heterosexuals (Björkenstam et al., 2016). Despite the importance of online communities and dating sites for non-heterosexuals and the relative progressiveness of LGBTQ issues in the Nordic countries (Svensson, 2016; Flores et al., 2018; Lagerberg, 2018; The International Lesbian Gay Bisexual Trans and Intersex Association, 2019; Timofejevs-Henriksson, 2011), there’s a shortage of research on the topic from a Nordic perspective. This perspective offers new insight to the subject as the Nordic countries, in addition to being viewed as liberal, in general have low population density with large rural areas (Eurostat, 2017). With this follows that the available *online* dating sites are of great importance for many Nordic non-heterosexuals. The scarcity of both off- and online alternatives, especially for non-heterosexual women, makes it all the more important to examine the leading online dating scenes for Nordic non-heterosexuals.

## The Non-Heterosexual Online Dating Scene

The LGBTQ scene is commonly seen as open-minded, inclusive and tolerant, but in reality sexism, misogyny, racism, homophobia and other forms of discrimination are in no way absent from the non-heterosexual world, which includes online dating (Connell, 1992; Phua and Kaufman, 2003; Wood, 2004; Ward, 2008; Miller, 2015; Robinson, 2016). The majority of the previous research on self-presentation among non-heterosexuals online has focused on men, while the lesbian online dating market continues to be framed as a problem by users, developers and investors (Murray and Ankerson, 2016). On mixed-sexuality sites, such as Tinder, non-heterosexual women experience a feeling of scarcity in relation to other women (Duguay, 2019). By rejecting a compulsory heterosexual way of life, lesbian existence has largely been found where lesbians have shared common cause with gay men, but lesbian existence in itself, and important differences between non-heterosexual men and women, have historically been neglected in research (Rich, 1980; Valentine, 2000; Wilkinson, 2008). Rich’s observation holds true today as considerably more studies have been conducted on online communities for non-heterosexual men compared to sites for non-heterosexual women or mixed-gender sites, in accordance with the historical gender-imbalance in sexuality research (Connell and Messerschmidt, 2005; Murray and Ankerson, 2016). The limited previous research that does include non-heterosexual women has shown gender-specific differences between non-heterosexual men and women, including variances in prevalence of disclosure of sexuality, choice of profiles pictures and aspects valued in potential partners and relationships (Hatala and Prehodka, 1996; Miller, 2015; Potârcã et al., 2015; Reynolds, 2015; Lemke and Weber, 2017). Another notable difference is that non-heterosexual men are significantly more likely to state racial preferences online compared to non-heterosexual women (Rosenfeld and Byung-Soo, 2005; Rafalow et al., 2017) and ethnic minority men are discriminated against to a greater degree than minority women (Lundquist and Lin, 2015). Online dating sites may be the only remaining social context where it in many cases still is deemed appropriate to announce one’s racial preferences (Lundquist and Lin, 2015). Numerous online dating sites encourage members to use simplified racial labels, both to describe themselves and as a preference search tool for potential partners (Callander et al., 2015). White non-heterosexuals online are less likely to exclude their own racial group compared to non-heterosexuals of color, which reflects the current racial hierarchy (Phua and Kaufman, 2003; Rafalow et al., 2017). Black non-heterosexual men are commonly placed in the lowest position on the racial hierarchy and are particularly subjected to sexual objectification on online dating sites (Teunis, 2007; Ward, 2008). Gender expectations and discussions about femininity and masculinity are also of great importance on online dating sites for non-heterosexual men, where a hypermasculine, sexualized ideal regularly is promoted (Ward, 2008; Boyd Farmer and Byrd, 2015; Tziallas, 2015). It is not unusual that these sites endorse pornographic self-presentation (Tziallas, 2015) and a quantification of bodies, with measures of height, weight and genitals, which promotes ideals of tall, fit bodies and discriminates against non-normative bodies (Robinson, 2016). In the present study, the gender scope is limited to men and women. The reasons for excluding transidentified users are presented under inclusion criteria. For most of us, biological sex characteristics and gender are aligned (cis-gender), while they are not aligned for transgender people. The un/alignment that constitutes cis-gender and transgender as discrete identities is based on a structure that installs sex/biology as having defining priority over gender/identity, where sex and gender is fixated in relation to the male/female binary (Detournay, 2019). This is seen in the Swedish trans-specific healthcare, where gender is still at large constructed as norm-conforming and binary (Linander et al., 2019) and in Swedish newspaper, where articles meant to empower trans people reinforce heteronormativity through constant referral to binary gender (Åkerlund, 2019). Similar to the United States, where the transgender movement successfully has changed United States public policy over the past two and a half decades (Nordmarken, 2019), acknowledgment, theory formation and discussions about trans and non-binary issues have increased in the Nordic countries (Haavind and Magnusson, 2005; Magnusson, 2011). Non-binary or gender fluid people do not limit themselves to one of the two established genders or stereotypical expectations of men and women (Gosling, 2018). The Swedish word *kön* (sex) signifies both the biological and social sex and does not refer to sexual practices, as the equivalent English word does (Liinason, 2011). To reduce the male bias in language, where the implicit belief is that a word describing an undefined person describes a man, a third-person gender-neutral pronoun singular (hen) has been introduced in the Swedish language (Lindqvist et al., 2019). Without disregarding recent productions of gender, the concepts “man” and “woman” where in the present study found to be useful analytic tools, especially motivated by the gender-imbalance in previous research on non-heterosexuals online.

## Self-Presentation

Goffman defined self-presentation as the way people constantly try to manage how others perceive them, by always playing roles when interacting (Goffman, 1959/1990; Attrill, 2015). His theory was presented during a time when there still existed a presumed arena where people could be themselves: home alone (Goffman, 1959/1990; Agger, 2012). With the Internet, which is easily accessible in most Nordic homes of today, and even more so through the everyday use of smartphones, the presentation of the self continues to endlessly be played out online. The online world penetrates what Goffman called the “backstage,” our private life, which changes the ways we relate to the self and self-presentation (Goffman, 1959/1990; Agger, 2012; Blackwell et al., 2015). As digital technology has become increasingly portable, we rapidly shift between online and offline interactions, blurring the lines between public and private spaces even more (Parisi and Comunello, 2016; Choy, 2018). The shift toward visual imagery, where interacting includes, or is made up of, photos and pictures also significantly affect our self-presentations (Jones, 2005). Self-presentation is always constructed and manipulated to fit temporal and situational norms and in online dating this is complicated by the fact that the framing of self is done for an *anticipated* audience (Agger, 2012; Attrill, 2015). In any given social context we respond to other people’s reactions to our self-presentation. This creates an interactive stage, where individuals and groups are working singularly or together to maintain impression-management of one another (Goffman, 1959/1990; Attrill, 2015; Nash and Gorman-Murray, 2019). Dating apps, especially those with geolocation services, bring excitement and opportunities but also tensions to self-presentations connected to identifiability and new and constantly changing norms (Blackwell et al., 2015). Goffman’s theory undermines the notion of authenticity that most people hold dear. Both off- and online, people must navigate the emotional dissonance of trying to be their true self and still manage others’ perceptions and interpretations of the self in a fashion that places them in a positive light (Suler, 2004; Attrill, 2015). Self-presenting online can foster a truer self-presentation compared to face-to-face interactions, supposedly facilitated by the absence of traditional gating elements that dominate initial relationship formation (Bargh et al., 2002). The level of authenticity both off- and online is affected by whether people expect to meet someone again. Both men and women display similar and higher levels of lying when they don’t expect to meet a new person again (Tyler and Feldman, 2004). In online dating, you never have to meet the person or audience you self-present for if you don’t wish to (Agger, 2012). Still, people generally report that they attempt to self-present truthfully in online dating profiles. However, this goal is often in tension with the natural inclination to frame a version of the self that is thought to be desirable (Ellison et al., 2006). The use of flattering profile photos is not surprising as individuals who represent dominant beauty ideals in society usually are in a better position to exploit a wider range of people on online dating sites (Hobbs et al., 2017). Non-heterosexuals online have been known to self-present in a less authentic fashion compared to heterosexuals, and here self-esteem seems to be the most important predictor in fostering authentic self-presentation (Ranzini and Lutz, 2017). Another reason for a stronger inclination for self-presenting a more fantasy self among non-heterosexuals is that the presented self sometimes simply cannot be expressed offline. Manipulation in self-presentations can also be done in order to avoid disapproval and to achieve a sense of belonging in the online community (Attrill, 2015). Thus, it is motivated to study self-presentations of non-heterosexuals online to examine what kind of self-presentations are perceived as successful in this longing for belonging.

## Aim

Previous research has in large been geographically specific to Anglo-Saxon countries and focused on male experiences and behaviors online (e.g., Clarkson, 2006; Ward, 2008; Callander et al., 2015; Reynolds, 2015; Robinson, 2016). The gaps in the literature concerning the present study’s main objective are considerable. When running database searches on Scopus and Web of Science, limiting the search scope to a Nordic setting, only a handful of articles were found. These articles were disparate in focus, ranging from factors associated with condom use and HIV testing (Johansson et al., 2018) to political discussions on a Swedish queer online community (Svensson, 2015). Even with the reservation of alternative search strings, it is safe to conclude that there exist literature gaps concerning self-presentations on the Nordic LGBTQ online dating scene. Building on international research the present study analyzed self-presentations among 716 cis-gendered, predominantly Swedish online dating profiles on a well-established Nordic online dating site for non-heterosexual men and women. The fact that the examined site is a mixed-gender site offers a rare opportunity to investigate the interplay of gender and sexuality as a majority of international dating sites exclusively target non-heterosexual men *or* women (e.g., Grindr, Scruff, and HER). Examining possible gender-based issues and differences in self-presentations from a Nordic perspective is especially motivated as the Nordic countries dominate global rankings of gender-equality (World Economic Forum, 2018). The findings in the present study should be revealing of how online self-presentation, gender and representation is manifested among non-heterosexuals of small, progressive countries, in addition to offering opportunities for critical discussion of the LGBTQ scene in general.

The main objective of the present study was to examine self-presentations on the Nordic online LGBTQ dating scene. The two research questions guiding the study were:

• Which central self-presentations exist on the Nordic online LGBTQ dating scene?
• What possible gender-differences are found in self-presentation on the Nordic online LGBTQ dating scene?

## Materials and Methods

### Participants

Data was collected from members of a large dating site that for many years has functioned as an established online community for Nordic LGBTQ people. The community is both a static desktop site and an app. The app does not provide geolocation service, highlighting other users’ geographic proximity, in the same way as e.g., Tinder or Grindr (Stempfhuber and Liegl, 2016). Membership on the site is free of charge. Mandatory information needed to create and activate a basic profile is gender, sexual orientation, age and a user alias. Photos and profile texts are optional. With the exception of illegal content, e.g., pedophilic content or content that could be regarded as hate crimes, there are no restrictions as to what kind of photo a user can upload, i.e., photos of genitalia and porn are allowed. Other users can choose to block these kind of profiles, on the site called “xxx-rated,” in their searches. However, it is not possible to block the profiles from browsing or contacting your profile. To gather data, a basic profile without text or photos was created. This profile had minimal appeal on the site with ten of thousands profiles, where most members demand photo to engage in contact. The created profile didn’t contact any members on the site and neither was it contacted during the duration of the study.

A stratified selection procedure for participants was conducted through two separate searches – one for women and one for men. Gallery setting was chosen, which meant that all included profiles had photos. On the starting date of data collection, 10886 women and 31770 men were members with profile pictures. Profiles were shown in alphabetic order. To ensure an even gender distribution and a representation of the whole alphabet, one woman on every page and one man on every third page was selected. The study used random.org to randomize what profile to analyze on every page, which in default mode displayed 30 profiles per page. The randomized profile was number nine on each page for women, and profile number 14 on every third page for men, which in total amounted to data from 716 profiles (363 women and 353 men), of which 503 members (252 women, 251 men) had a profile text. The youngest member was 16 years old and the oldest 77 years old. The mean age for the whole sample was 36 years (for women *M* = 32, *SD* = 13 and for men *M* = 40, SD = 9). Four hundred eight members (227 women, 181 men) presented themselves as single, 157 (80 women, 77 men) as being in some sort of relationship, and 151 members didn’t reveal their marital status.

### Variables

On the selected site, members can self-present and search for other members via several pre-programed variables such as sexual orientation, ethnicity, age etc. Quantitative variables collected in the present study were age, gender, ethnicity (on the site named” origin of looks”), sexual orientation, and marital status. While age, gender and sexual orientation are variables mandatory to state, ethnicity and marital status are optional.

Data was also collected regarding the random profiles’ choice of alias, profile picture and profile text. Alias were categorized as “sexual” and “non-sexual,” where the first comprises aliases with sexual references, e.g., to genitalia, sexual acts and sexual preferences, and the latter to all other aliases such as personal names and literary, cinematic, or nature references. This coding was by necessity subjective, with the main “risk” being that aliases with sexual slang unfamiliar to the author have been coded as “non-sexual.”

Profile pictures were categorized as “pornographic and/or genitalia,” “nude body,” “full-length (dressed),” “face picture,” and “neutral motif.” The profile pictures were additionally categorized as identifiable (recognizable face photos) or non-identifiable (genitalia, blurred face pictures, nature photos etc.). Profile texts were saved in a separate word document and used in the qualitative analysis.

### Inclusion Criteria

Inclusion criteria are cis-gendered, Swedish profiles with a profile picture. The scope of the study was limited to sexuality. Trans-identified members (signaled by a t behind gender in their profiles), regardless of sexuality, were excluded. One reason for this was that the author did not feel comfortable labeling the minority of the users who stated a t in their profile. The users had to choose a gender (man or woman) as there didn’t exist a third option/gender. Therefore, the use of t could indicate anything from fluidity or non-binary to transvestite or transsexual identity. The author did not have enough information to determine how this small fraction of members gendered, or dis-gendered themselves. Owing to the random sampling method used, the number of trans-identified profiles in the present study would have been so small that no valuable analysis or conclusions could have been established. Research questions concerning gender identity are in conclusion considered to warrant a study of their own, preferably a study where random sampling is not used. Profiles for couples, typically a man and a woman, were likewise excluded. If the randomized number on any given page showed a trans-identified person or a couple, profiles one step ahead or behind (randomized) were used instead. Members who, with a site-provided flag symbol, signaled that they did not live in Sweden were excluded owing to Swedish ethical approval regulations. The majority of the dating site’s members supposedly were Swedish and the main groups excluded through this strategy were residents living in Finland, followed by Norway. The same procedure as with trans-identified and couples profiles was used when a randomized profile signaled that they were not Swedish. Stating country of residence is however, optional on the site. Also, nearly a third of the selected profiles had no profile text and therefore many provided no linguistic clue about country of residence. In sum, unintentional inclusion of members who live in the other Nordic countries or elsewhere is likely.

### Thematic Analysis

Thematic analysis (TA) was used to analyze the profile texts. TA is a widely used method within psychology and is by the general public an easily understood form of qualitative analysis (Braun and Clarke, 2006; Riessman Kohler, 2008; Howitt, 2016). TA is known for its flexibility in regard to theory, research questions and data collection methods and as the name suggests, TA involves the recognizing, and analyzing of major themes found in qualitative data (Willig, 2013; Howitt, 2016; Clarke and Braun, 2017). Any form of textual material can be used in TA, including material from the Internet, where the constantly changing ways of online communication constantly produce new forms of data (Flick, 2014; Howitt, 2016). TA can be used for both inductive and deductive analyses, capturing both manifest and latent meaning (Clarke and Braun, 2017). The present study used inductive TA, coding the data without trying to fit it into preexisting theories, whilst not losing awareness of the fact that we always carry theoretical presumptions (Braun and Clarke, 2006). A theme in TA captures something important about the data in relation to the research questions (Braun and Clarke, 2006; Howitt, 2016) and refers to a specific, recognizable pattern of meaning, which co-occur in a meaningful and systematic way rather than random and arbitrary (Willig, 2013). TA requires an intimate knowledge of the data (Braun and Clarke, 2006; Willig, 2013; Howitt, 2016), which in the present study was achieved by reading all profile texts, arranging them in word documents and re-reading them multiple times through the analytic process (Howitt, 2016). Braun and Clarke’s (2006) systematic and accessible procedure was used during the analysis, following the six phases that the authors have outlined. Phase 1 – familiarization with the data – was accomplished through reading and re-reading the profile texts and noting down initial comments. In phase 2, the more formal coding process took place, generating preliminary codes from the initial list of ideas scribbled down in phase one. The codes identified potential interesting features of the data. As coding does not have to be done line-by-line (Howitt, 2016) coding was conducted for sentences or short profile texts in their entirety when advantageous. The entire data set was systematically worked through, giving equal attention to each data item to identify aspects in the data that formed basic repeated patterns (Braun and Clarke, 2006; Howitt, 2016). Coding was done for as many potential themes as possible, where individual extracts of data were coded into several different themes when relevant. In phase 3, the list of different codes and relevant extracts were sorted and used to analyze how different codes together formed possible overarching themes and sub-themes within them. Refinement of the candidate themes followed in phase 4, reviewing which themes really could count as themes, which themes needed to be collapsed with other themes and which themes might need to be broken down into several themes. Establishing identifiable differentiation between themes and coherence of data within themes was the goal of the reviewing and rewriting of themes in phase 4 (Braun and Clarke, 2006; Howitt, 2016). In phase 5, further refining and naming of the themes took place, identifying the “essence” of each theme. In the sixth and final phase the final paper was produced. As customary, quotations are embedded in the analysis to illustrate the identified themes. Selected quotations have in some cases been abbreviated to focus on the theme currently under examination and are translated from Swedish to English, re-translated, and in some instances linguistically corrected to erase dysfluencies and unnecessary grammar misunderstandings (Riessman Kohler, 2008).

### Ethical Considerations

The Internet is a tool, a social phenomenon, a communicative venue and a field for research. Unsurprisingly, internet-mediated research (IMR) raises particular ethical challenges (Markham and Buchanan, 2012; Hewson and Buchanan, 2017; Buchanan and Zimmer, 2018). A recurrent topic in online ethics is whether data from e.g., social media is to be considered public or private (Flick, 2014). It is debatable if there can be any reasonable expectation of privacy in the ongoing era of ever-present online surveillance and data tracking (Markham and Buchanan, 2012; Buchanan and Zimmer, 2018). The data used in the present study cannot be re-identified as the level of linkability of data to individuals, and thus the potential harm such disclosure could pose is extremely low (Buchanan and Zimmer, 2018). Since no data that could lead to a directly or in-directly identified or identifiable person was used, the regulation concerning special categories of personal data is regarded non-applicable. Publishing the name of the dating site might have negative effects on the online community (Hewson and Buchanan, 2017), which is why this information is not included in the present study. The face photos are the only data that hypothetically could be used to recognize a living person from, although the risk that the author would stumble upon someone recognizable was considered slim and indeed never happened. Before data collection, it was decided that should such an instance have occurred the profile would not have been included. The categorization of profile pictures was done directly from the site and no photos were downloaded or saved. On the site, members can search for other members by their alias, which is why aliases were directly categorized and then saved in a separate document for possible necessary second-opinions concerning the coding. The sources of the quotations from the profile texts cannot be found by searching the entire quotation or segments of it on the dating site, as the site doesn’t allow text search. The present study did not obtain informed consent from the random sample. Seeking informed consent from deliberately anonymous online profiles was decided to be a sub-optimal ethical strategy, as the online members then would’ve be forced to disclose their identity. In accordance with the dating site’s guidelines, all members use pseudonyms and might never be known beyond their screen name, and the present study did not wish to disrupt this space of privacy. Possible psychological, economic, or physical harm to the online dating site’s member’s lives, careers or reputations, that might occur in research where there’s a risk of “outing” an LGBTQ individual (Markham and Buchanan, 2012) is, thanks to the tremendously low level of traceability, reduced to minimal in the present study. The scientific and social value of the present research is deemed large enough to justify the undisclosed observation. Ethical approval was applied for in Sweden and the ethical board in Umeå concluded that the present study was not categorized as regulated under ethical review.

## Results

In Table 1, frequencies of sexual orientation are presented in the same order as originally provided on the site. It is unclear why heterosexual is the first option on the LGBTQ dating site. As would be expected, a majority of the site’s members identify as non-heterosexual, with homosexual, bisexual and queer amounting to 84% of the population. There exist gender-differences in sexual orientation, Chi^2^ (6) = 38.21, *p* < 0.001 (Cramer’s *V* = 0.23) with more women presenting as bisexual (standardized residual, *se*_*i*_ = 2.2) compared to men (*se*_*i*_ = 2.2), while men more often present as experimental (*se*_*i*_ = 3.2) compared to women (*se*_*i*_ = 3.1).

**TABLE 1:** Frequencies – sexual orientation (expected frequency within parentheses).

**Sexual orientation**	**Men**	**Women**	**Total**
Heterosexual	13 (9.4)	6 (9.6)	19
Homosexual	188 (181.4)	180 (186.6)	368
Bisexual	83 (106.0)	132 (109.0)	215
Queer	6 (9.9)	14 (10.1)	20
Experimental	52 (33.5)	16 (34.5)	68
Asexual	0 (1.0)	2 (1.0)	2
Other/don’t know	11 (11.8)	13 (12.2)	24
Total	353	363	716

In Table 2, frequencies of ethnicity for the 496 members (231 women, 265 men) that choose to disclose ethnicity/“origin of looks” are presented in the same order as originally provided on the dating site. There are no significant gender-differences in presented ethnicity, Chi^2^ (10) = 10.01, *p* > 0.05. However, significantly more women (*se*_*i*_ = 1.9) than men (*se*_*i*_ = 2.0) choose to *not* enter information about ethnicity, Chi^2^ (1) = 10.99 *p* < 0.001 (*Phi* = 0.12).

**TABLE 2:** Frequencies – ethnicity (expected frequency within parentheses).

**Ethnicity/origin of looks**	**Men**	**Women**	**Total**
North European	223 (216.4)	182 (188.6)	405
West European	7 (11.8)	15 (10.2)	22
Central European	3 (3.2)	3 (2.8)	6
South European	6 (5.3)	4 (4.7)	10
East European	1 (3.2)	5 (2.8)	6
Asian	6 (5.3)	4 (4.7)	10
Indian	2 (1.6)	1 (1.4)	3
African	1 (1.1)	1 (0.9)	2
Middle Eastern	4 (3.2)	2 (2.8)	6
South American	5 (6.9)	8 (6.1)	13
Other	7 (6.9)	6 (6.1)	13
Total	265	231	496

### Alias

A total of 627 members (356 women, 271 men) have a non-sexual alias and 89 members (7 women, 82 men) have a sexual one (generic sexual aliases: fuck me hard, big dick slut). There are significant gender-differences, Chi^2^ (1) = 74.60, *p* < 0.001 (*Phi* = 0.32), with more men (*se*_*i*_ = 5.8) presenting with a sexual alias compared to women (*se*_*i*_ = 5.7), who instead more often present with a non-sexual alias (women *se*_*i*_ = 2.1, men *se*_*i*_ = 2.2).

### Profile Pictures

In Table 3, frequencies of type of profile picture are presented in the order of the self-created five-step categorization of profile pictures, from pornographic to neutral. There are significant gender-differences in the type of profile picture used, Chi^2^ (4) = 200.67, *p* < 0.001 (Cramer’s *V* = 0.53), with men more often using genitalia/pornographic pictures (men *se*_*i*_ 7.4, women *se*_*i*_ −7.3), nude pictures (men *se*_*i*_ = 3.8, women *se*_*i*_ = 3.7), and full length body pictures (men *se*_*i*_ = 2.1, women *se*_*i*_ = 2.0), while women (*se*_*i*_ = 5.3) more often use face pictures compared to men (*se*_*i*_ = 5.3).

**TABLE 3:** Frequencies – type of profile picture (expected frequency within parentheses).

**Type of profile picture**	**Men**	**Women**	**Total**
Genitalia/pornographic	104 (51.3)	0 (52.7)	104
Nude body	42 (23.7)	6 (24.3)	48
Full length body - dressed	27 (18.2)	10 (18.8)	37
Face	159 (242.1)	332 (248.9)	491
Neutral (nature etc.)	21 (17.7)	15 (18.3)	36
Total	353	363	716

### Identifiable/Non-identifiable

Members with a clearly identifiable profile picture, which by necessity implies a *face* photo without blurring, gigantic sunglasses or other face obscuring features, were coded as identifiable, while the rest were coded as non-identifiable. Four hundred and forty-one members (303 women, 138 men) were coded as identifiable and 275 (60 women, 215 men) as non-identifiable. Once again there exist a significant gender-difference, Chi^2^ (1) = 148.99, *p* < 0.001 (*Phi* = 0.46), with more women (*se*_*i*_ = 5.3) being identifiable compared to men (*se*_*i*_ = 5.4).

### Mind and Body

In the thematic analysis, three main themes were identified, composed by three subthemes each. Before embarking on the themes, it should be noted that an individual profile text might contain several themes. The first main theme “*Mind and body*” includes the subthemes “*mind as personality*,” “*the body*,” and “*genitalia and identifiability.*” In this theme, the pervading pattern is a gender-dependent emphasis on one hand on the mind and personality and on the other hand on the body and physical assets. Naturally, there exist overlaps, where especially men self-present through *both* mind and body. The concepts used in the findings were generated through the analysis – not operationalized in advance. However, “mind” needs some conceptualization. In the most simplified summary possible, the “mind” is in the present study conceptualized in accordance with the following OED definition: “The mental faculty of a human being (esp. as regarded as being separate from the physical); (occasionally) this whole system as constituting a person’s character or individuality.” The mind is metaphysical in nature, and mind-body, or mind-brain, correspondence and definitions continues to be a challenge in 21st century psychology (Bell, 2002; Barrett, 2009). Complex psychological categories such as “mind” are in the end always observer dependent (Barrett, 2009).

#### Mind as Personality

In the first subtheme, the mind is in the present context generally framed as personality. Self-presentation through personality is on the selected site done by depicting personality in three main fashions – from altogether positive to downright negative or sarcastic. It is hardly surprising that a majority of members choose to market themselves as having a positive personality (Ellison et al., 2012; Attrill, 2015). Recurrent positive personality wording for women is “happy” and for men “nice.” Below a quote from a woman, followed by a quote from a man, both in their late thirties:

*A happy*, *helpful*, *good-hearted woman*, *who usually prioritizes other people./*…*/I love having coffee with my good friends*, *snuggling up in the sofa to watch a movie*, *listen to music*, *sing etc.*

*Hi! I’m a social and nice guy who enjoys classical music*, *parties*, *orchids and all good things in life. Please write something nice.*

Personality is closely linked to interests and vice versa. With the exception of sexual interests, no notable gender-differences in interests are found. Some members react with humor to the positive personality trend, presenting themselves in styles similar to this man in his mid-thirties:

*I guess I could write an essay here about how wonderful and lovely I am*, *but I think it’s more up to you to discover than for me to suggest.*

Most online dating users know that a slight misrepresentation, through highlighting or exaggerating of positive characteristics, is both “allowed” and expected. This is rationalized by the notion of multiple selves from a wide temporal spectrum, and the characteristics the user self-presents with are only deemed unacceptable if the discrepancy between the “real” self and the presented self is too large to bridge (Ellison et al., 2012). The positive framing of personality is however, far from the only fashion of self-presentation connected to the mind. Almost as common is the “nuanced” personality, where frequent wording instead include “shy” and “nerdy.” Here, positive perfection is not pursued. Instead, a varied personality is presented:

*Are you like me: completely imperfect*, *pretty nice and down to earth – then it’s a good start!/*…*/man in his early twenties.*

*I’m a happy but somewhat shy gal*, *taking a big interest in life. I’m not cool or hip. I’m pretty shy with new people*, *but once I get to know someone I’m very open/*…*/woman in her early twenties.*

In general, nuancing the self-presentation could assumingly facilitate moving from chatting online to meeting in real life. If a person’s intent is to date offline, manipulating the self-presentation, whether focused on mind and/or body, in a way that creates a too noticeable discrepancy between on- and offline impression is probably not the best strategy (Ellison et al., 2012; Attrill, 2015). Lastly, we have the members who self-present by including personality characteristics that by societal standards usually are not regarded as desirable. Here “qualities” such as a bad temper, different diagnosis and a general pessimistic outlook on life are presented:

*I’m a loner and wiseacre*, *who spend all my money on cats and alcohol. On sick leave*, *bipolar*, *ADD etc. – you know the drill. Breeder of cats*, *Cornish rex. Anything else you need to know? Well*, *then send me a message./woman in her early twenties.*

*I have poor balance*, *seldom shower and am both a bad winner and a bad looser. Leftist*, *feminist*, *vego. Fuck the cistem*, *no cake for the upper-class*, *etc./woman in her thirties.*

Many of the “negative” personality self-presentations use humor or sarcasm as a trademark. In the above quote the woman is presumably being self-deprecating while at the same time including more factual identity markers such as political standpoints. It seems likely that this is a political conscious woman who with “fuck the cistem” is critical of majority society’s gender notions and constructs, even if she doesn’t necessarily (at least not by the provided t label) identify herself as trans or non-binary. Presenting ‘negative’ personality qualities is not as prevalent among men and when it does occur it usually quickly moves to something more body-oriented:

*Odd*, *special*, *fat – chunky*, *looking for other fatties/softies between 20 and 35*, *a plus if you’re passive/*…*/man in his fifties.*

#### The Body

With the above quote, a transition to the second subtheme “*the body*” is provided. Here placed importance on the body, measures, demands, and insecurities are examined. Balancing on the borderline of mind and body, (hyper)masculinity has dominated gay male ideals and representation (e.g., Connell, 1992; Brennan et al., 2013; Nash, 2013), with a spillover effect in self-presentations online (Wood, 2004; Brennan et al., 2013). Indeed, preferences concerning masculinity have been found to be the most frequently discussed topic in non-heterosexual men’s online profiles (Rafalow et al., 2017). This is not the case in the present study. Nonetheless, masculinity is present as one of many variables related to bodily self-presentations, with men highlighting their manliness in various ways:

*Curious guy*, *who joined to try something new. I’m tall*, *thin*, *brunette with blue eyes. I’m a manly guy*, *who now and then enjoys being fucked hard – if I’m logged in it’s one of those days/*…*/man in his mid-twenties.*

The above kind of straightforwardness and (over)sharing of body and intimacy is facilitated by the fact that the sharing can be turned on and off instantaneously online (Suler, 2004; Agger, 2012; Blackwell et al., 2015; Choy, 2018). It is also encouraged by the fact that every self-presentation is in competition with an incalculable number of others for an anticipated always-present audience (Jones, 2005; Parisi and Comunello, 2016; Hobbs et al., 2017). Yet, this does not explain why women in general do not “seize the opportunity” and self-present in a similar fashion. This discrepancy might instead be attributed to gender norms within and beyond the selected online community. Femininity and feminine women have been shown to occupy the top of the hierarchy of desirability on online dating sites for non-heterosexual women (Farr, 2011; Hightower, 2015). However, on the selected site overt discussions about masculinity or femininity is basically non-existent in the women’s self-presentations, with some rare exceptions:

*Chat or sex with younger*, *same-age*, *older girls/women or guys/men (feminine*, *girlie*, *dominant*, *active)/woman in her thirties.*

Similar to previous research findings (Robinson, 2016; Miles, 2019), a focus on measures – self-presenting in centimeters and kilos – is often manifested in the men’s profiles, while it is a non-subject among the women. Self-presenting solely with measures is not common among the men either, who instead complement stated measures with other characteristics:

*Hi! My name is Nick*, *I’m 25 years. I’m a teddy bear. I’m looking for a boyfriend to share my life with! I have a driver’s license/*…*/My measures are 196 cm*, *103 kg*, *15 cm. Feel free to write*, *I usually answer/man in his mid-twenties.*

While many men are preoccupied with body-oriented self-presentations, women generally meet the body with silence. Though the body rarely is given attention in the women’s profiles, some women stress a depiction of the body as a rather un-important issue. In such statements, a reconnection to the importance of the personality in potential partners is emphasized:

*It may seem* “*cheesy*” *but I don’t think looks are everything – it’s the inside that makes a woman become everything!/*…*/woman in her thirties.*

*Age*, *looks*, *weight*, *femme*, *butch etc. is unimportant for me*, *who believes that personality and the inside is what counts*, *and if we’re compatible/woman in her mid-fifties.*

#### Genitalia and Identifiability

In the third subtheme, “*genitalia and identifiability*,” displays of genitalia and discussions of identifiability are emphasized. Connected to “the body,” a recurrent genitalia-focus is found, where the own presumably large penis and/or desires about so called well-equipped men is highlighted:

*185/81/17 looking to meet a man*, *preferably 60* + *for sex. I enjoy fondling/necking/jerking off/sucking. I love cock sucking*, *a plus if you’re well equipped/man in his sixties.*

Both the textual and visual commonly occurring focus on genitalia in men’s profiles in part confirms previous research about online dating sites for non-heterosexual men being steeped in, and promoting, a highly sexualized culture (Valentine and Skelton, 2003; Tziallas, 2015). In the present study, not all the site’s members appreciate the sexualized self-presentations. Seemingly having met disapproval from fractions of the site, some men comment on their display of genitalia:

*Sorry about the dic pic but my face will probably not end up on this site. I’m an inexperienced guy*, *curious to try most things/man in his forties.*

Apologetic or not, every man who mentions their nude photos is more or less blunt about not wanting to be identifiable. A key attribute of self-presentation in any context is control over one’s identifiability (Blackwell et al., 2015). Among non-heterosexual men in the online dating context the most common way to protect anonymity is to offer a picture of the body but not of the face (Jones, 2005), a method used by men in the present study. The decision to be identifiable or not is influenced by the user’s visibility as a non-heterosexual person in everyday life (Parisi and Comunello, 2016). There is less emphasis on anonymity and a total lack of discussion of genitalia in the women’s profiles, with the exception of women trying to safeguard themselves against male genitalia (see theme 3). Amongst the men’s bodily self-presentations, departures from the ostentatious and factual exist. Insecurities and self-criticism, usually connected to age and/or weight, are displayed:

*I’m looking for a younger*, *fresh*, *well-shaped active man who’s turned on by older*, *passive men like myself. I’ve lost some of the well-shaped*, *slim body and now have a prominent big belly and my dick has never been big*, *but everyone thinks I’m nice and social/*…*/man in his seventies.*

Self-consciousness about the body is logical as young, fit bodies are promoted as ideals within the gay scene (Robinson, 2016). Interestingly, perceived shortcomings concerning the own body are commonly *not* correlated to what is desired in others, as can be seen in the above quote. Staying non-identifiable could, in addition to the aforementioned reasons, reduce the risk of rejections of the displayed body being perceived as (as) personal.

### Framing Lust and Longing

In the second main theme, “*framing lust and longing*” the sub-themes are “*lust*,” “*domination and location*” and “*longings for love.*” In this theme, a mind versus a body emphasis continues to act as the underlying structure and gender once again as the common denominator. In framing lust and longing a wide rage of human desires are incorporated, from pornographic fantasies to dreams of life-long love.

#### Lust

In the first subtheme, lust is in focus. Once again, some degree of conceptualization is warranted. Departing from the OED the author similarly defines lust as “sexual appetite or desire” and “the passionate desire *of* or *for* some object,” without the biblical and theological connotations of lust as “sinful or leading to sin.” On the selected site, lust is discussed on a continuum of straightforwardness, but in general the men’s framing of lust doesn’t leave much to the imagination:

Looking for a guy who wants to fuck my tight ass/man in his forties.

While women very seldom mention lust and sex, it is a recurrent and prominent feature in many men’s profiles, often presented in the above blunt manner or in more detailed accounts of explicit sexual fantasies:

*Welcome home to my apartment for sex. Come to me with your cock. I’m naked*, *watching porn yearning for cock visits. I have a nice suck mouth/*…*/I won’t say no to multiple cocks at once. If I’m logged in*, *I want one or several cock visits/man in his seventies.*

The identified focus on sex and lust is not surprising. Rather, it is in line with previous research, where sex has been found to be one of the most important motivators for non-heterosexual men on gay dating apps, which are viewed as hospitable to taboo talk (Gudelunas, 2012; Parisi and Comunello, 2016). Non-heterosexual men also indicate strong support for the positive aspects of sexual explicit material (porn) (Lewis et al., 2018). In line with previous research about the hypermasculine ideal leading some men to act “no homo” (Wood, 2004; Nash, 2013), many men in the present study frame their sexual desires as a mere complement to their heterosexual sex life, as something very rare or as something new:

*I love sex but have only had it with women. I want to meet a sexy*, *feminine trannie to have uninhibited sex with. I’m good looking and can’t wait to please you! To fuck a guy in women’s clothes is a dream I’ve had for a long time/*…*/man in his sixties.*

Online dating can easily incite a shopping-oriented mindset, leading users to objectify each other (Jones, 2005; Finkel et al., 2012) and sexualizing of the femininely coded trans body is common (Åkerlund, 2019). Even though some men, as previously mentioned, choose to highlight their masculinity, presentations of lust for men in women’s clothes is not uncommon. Supposedly, a high number of men on the selected site desire transidentified men and/or perhaps it is a way for some men to more effortlessly slide over to homosexual experiences. Closely linked to men’s general presentations of sexual desires is the frequent emphasis on discretion:

*I’m a serious man looking for a serious*, *discreet fuck buddy/man in his fifties.*

Demands of discretion go hand in hand with the above-mentioned wish for anonymity.

#### Domination and Location

In this second subtheme, lust is presented along a sliding scale of submission and domination. Women presenting dominant or submissive desires are a rare occurrence compared to men. Below, one of the few female accounts about desiring domination:

*What I’m looking for here are dominant women who know what they want and who can guide a curious woman. Preferably a combination of friendship and benefits/*…*/woman in her thirties.*

Some men take domination and submission one-step further, with self-presentations including the usage of words such as ‘servant’ and ‘slave’. The following quote is from a man who self-presents through fantasies about total humiliation:

*Looking for a very stern Master! Seeking a master who wants a slave and not a boyfriend*, *no cuddling or friendly talk instead total ownership*, *humiliation and control/*…*/man in his late forties.*

Framing lust is context-dependent, to the site and beyond, which some members choose to highlight. One often-mentioned factor in this specific context concerns marital status. Again, the “straight-acting” self-image adapted by some men presents itself, where lust for men is portrayed as a sidetrack from their “real” heterosexual lives:

Am in a relationship with a woman but sometimes crave cock. Recently discovered that there’s no better feeling than a when a cock slides in. Love it!!!/man in his mid-thirties.

Some men in heterosexual marriages or relationships seem to have met a degree of disagreeability from other members, or predicted that this could happen. Below a defensive statement by a married man:

*Married man of 56 years*, *who is looking for sex on the side. Leave my page and spare me your moralizing if it bothers you. I only live once and don’t want to die curious. If you like sexual meetups irl*, *read on*… *Looking for a guy/man with really big/extremely big cock/*…*/man in his mid-fifties.*

Discussing marital status is considerably more common amongst men, in statements similar to the quotes above or in more implied accounts, with lines such as “can’t meet at my place if you know what I mean.” Non-heterosexual women generally give more importance to monogamy than non-heterosexual men (Potârcã et al., 2015). “Spicing up family life” is however, not an exclusively male topic:

*Woman of 44 years*, *checking out this site to get some excitement in everyday life. I’m bored both at work and in my marriage and am looking for men and women to experience adventures with/woman in her mid-forties.*

#### Longings for Love

In the third sub-theme, other framings of desires and dreams are found. Longings for love have not died online (Rosenfeld and Byung-Soo, 2005), and, indeed, for many members on the selected site a longing for friends and/or love is the main theme. Even though some men self-present with a declared wish for companionship, it is more common amongst women, who also are less hesitant to display longings for love in the “love of life” sense:

*I’m a teacher and love music*, *writing*, *wine and exploring what makes me feel. Looking for the woman*, *with a capital W*, *to share my life*, *love and everyday with*, *all that ordinary stuff/woman in her late twenties.*

For men, longings for love somewhat drowns among the more sexualized profiles, hence loosing visibility. Some men react with disappointment to what they perceive as a general lack of emphasis on love:

*Whatever happened to love and the desire to have a beautiful*, *shared and faithful life together with the person you would go to great lengths for?!/man in his mid-thirties.*

### Guarding Boundaries

In this last theme, members self-present by emphasizing resistance against different boundary breaking behaviors on the site. The sub-themes are “sexual harassment,” “age and ethnicity” and “disrespecting women.” Once again gender works as a crucial variable and in this theme, it’s the women who dominate.

#### Sexual Harassment

Women repeatedly use their self-presentations to attempt to protect themselves from unwanted sexual harassment by men on the site:

*BTW*, *if you write sick stuff like* “*do you want to suck my dick?*”*- Don’t even bother writing. I only get disgusted and irritated/woman in her twenties.*

In this subtheme it becomes evident that women on the selected site have experienced sexual harassment from men, often multiple times:

*I want to receive the same respect here as in life in* “*general.*” *If Ragnar*, *43*, *comes and throws his enormous*, *incredible hard and hairy dick in my face I would crush it*… *I want to do the same with all* “*Ragnar 43*” *here. So cut it out! Over and out/woman in her mid-twenties.*

It is troublesome that women have to use their self-presentations on a dating site for non-heterosexuals to fight off unwanted sexual attention from men. Unfortunately, it is not uncommon that men contact and harass non-heterosexual women on online dating sites, even when the women have set their settings to only show women and clearly signal their (homo)sexuality (Duguay, 2019; Ferris and Duguay, 2019). Men are not spared textual and visual sexual harassment on the present site. They too receive unsolicited texts and photos and try to guard against it:

*If you’re sensible – write! But*…*we probably don’t have much in common if you send a photo of a large asshole before you’ve even said hi*, *or have texts on your profile about laying ready with an open door for anyone to come and cum in you/*…*/man in his forties.*

Profile pictures are a crucial part of how people self-represent in online dating. The use of face-absent profile photos is a common feature among non-heterosexual men (Jones, 2005; Miller, 2015; Parisi and Comunello, 2016; Lemke and Weber, 2017), which is confirmed by the present study’s statistical findings and the many textual reactions against face-less members. Some men use humor or irony in trying to escape unwanted contact from nude, face-less profiles:

*Hit me up with an email if you want to know more about me./*…*/I adore sexual invites. Especially from middle-aged men without the slightest touch of self-criticism. Preferably without a face pic*, *instead exposing a short*, *wrinkly dick beneath a hairy potbelly/man in his late thirties.*

#### Age and Ethnicity

In this second sub-theme, boundary-building variables concern age and ethnicity. Men more commonly demonstrate this boundary. It is ambiguous if age boundaries should be regarded as a form of self-protection or as a sign of ageist ideals. Of course, both can co-exist on the site. Old age is known to be a disadvantage for participating in Swedish non-heterosexual dating (Siverskog and Bromseth, 2019). At the same time, young men often attract a lot of sexual pressure and attention on the non-heterosexual dating scene (Valentine and Skelton, 2003). It is possible that the following young man’s type of “harsh” self-presentation illustrates unwanted and repeated contact from older men:

*Hey! Homosexual guy*, *17 years old. NO OLD MEN!*

In limited instances women focus on age in guarding their boundaries. Below a woman displays her dismay in people of unwanted age groups (and men) contacting her:

Not interested in men or anyone younger than 30 or older than 50. Please respect this. NO WOMEN UNDER 30 OR OVER 50. Is that so hard to grasp?/woman in her late-thirties.

Despite the selected dating site’s policies that prohibit racism, anti-racism is also manifested as a boundary in self-presentations. Ethnicity is not commonly openly discussed, with the exception of a few men who exotify certain ethnicities, in line with previous research (Rafalow et al., 2017). Nevertheless it is safe to conclude that people, as demonstrated in other studies (Callander et al., 2015), are subjected to racism on the present site and therefore construct boundaries against it:

*Hi guys! I’m from the place where the Mediterranean*, *Middle East and Balkans converge. So if you are a racist bastard*, *don’t even dare/*…*/man in his late twenties.*

*You who are racist*, *Nazi*, *fascist*, *judgmental or overly religious*… *go on*, *get out and be gone. I’M NOT A WANDERING SLAVE OR SEX TOY*, *OK!?!/woman in her twenties.*

Racism and sexism intersect with one another and produces exclusion that impacts both body image and overall well being among racialized non-heterosexuals (Brennan et al., 2013; Brown, 2014). The identified anti-racism boundaries are in line with previous research showing that Nordic LGBTQ online communities are far from free from racism (Shield, 2016; Svensson, 2016).

#### Disrespecting Women

The last sub-theme, “*disrespecting women*,” deals with the most common guarding of boundaries, revolving around women trying to protect themselves against unwanted contact from men. One strategy used by women to safeguard is to simply, but firmly, point out that contact is exclusively sought from women. Another strategy is seen in the underlining of that which is *not* sought. Examples of both strategies can be seen in the three following quotes from women in the age range of 30 – 45 years:

I’m only looking for leastwise-potentially serious contacts with GIRLS and only GIRLS.

*What I’m NOT interested in is any form of sexual relation that has anything to do with any kind of man*.

*I reject dicks in all shapes and forms*, *don’t even bother.*

Some women incorporate additional information in the factual approach, explaining what lesbian means and/or humoring the men who don’t understand or respect the concept of their sexuality:

*The thing is that I like women. You men probably know that there exists setbacks in life*, *and I’m one of them./woman in her mid-twenties.*

The fact that men continually contact women on the site is so commonplace that a significant fraction of the women guard their boundaries with outright anger:

*GUYS*… *how overly explicit do I need to be?! NO!!! Don’t write to me*, *don’t flirt with me. Do NOTHING on this page!!!/woman in her thirties.*

*Guys - I will be a total ass to you*, *because some of you can’t read or just don’t want to read - If you message me*, *I will unleash hell on you. So piss the hell off/woman in her mid-thirties.*

Women’s inclination to self-present and interact will likely decrease in spaces where they clearly risk being harassed and unwillingly sexualized (James et al., 2019), which in conclusion is a widespread phenomena on the present Nordic LGBTQ dating scene.

## Discussion

Answering the research questions, the central self-presentations identified on the selected dating site were related to mind-body, lust-longings, and boundaries. Mirroring the quantitative findings, there existed pervading gender-dependent differences in all three themes. The fact that men dominated bodily self-presentations, through engaging in descriptions of the body’s features, functions and measures, resembled the statistical gender discrepancy in usage of sexual and nude photos and the use of sexual aliases. In theme two, women generally highlighted longings for love and companionship but seldom self-presented with lust, while this was a recurrent and prominent feature among men, who also often emphasized discretion. The quantitative findings supported men’s written desire for anonymity in the use of profile photos, as men significantly less frequently had identifiable photos. In the last theme, both women and men used different strategies, including facts, humor and anger, when trying to guard against unwanted sexual invites from male profiles.

The online dating scene operates on the basis of compartmentalized presentations of self-marketing (Finkel et al., 2012) and it is compelling, common and easy to modify the truth about yourself in online self-presentation (Suler, 2004; Agger, 2012; Ellison et al., 2012). Self-presentation online includes a “target” audience of multiple potential partners at any given time, in an atmosphere of marketing competitiveness, affecting self-presentations (Jones, 2005; Hobbs et al., 2017). While some of the users actively seek a partner of some kind, others are likely so comfortable in the online space that they have no desire to progress into off-line encounters (Miles, 2019). In the present study, neither authenticity nor actual intentions of the profiles are known. Consequently, the above-examined forms of self-representation are ultimately simplified portraits of human beings and the identified themes do not claim to be telling the “truth” about sexual minorities online. The author wishes to stress that even though significant gender differences in self-presentations were found in both the quantitative and qualitative data, the results are not intended as evidence of “natural” gender differences or of stagnant masculinity and femininity within the Nordic LGBTQ online community. No single identity category, such as gender, can satisfactorily explain a person’s behavior in different social settings (Magnusson, 2011; Geist et al., 2017). Nevertheless, the findings say something about what kind of self-presentations are normalized and dominating on the Nordic LGBTQ online dating scene. Self-presentation is always affected by the characteristics of a given social setting, encouraging individuals to choose identities in accordance with the context (Zhao and Martin, 2008; Magnusson, 2011). Like all places and spaces, this is a scene tied to identity categories such as gender and ethnicity and multiple power relations (Nash and Gorman-Murray, 2014). Obviously, the online dating scene is not detached from surrounding cultural norms and social values. Instead, these are often reinforced and played out online. Misrepresentation and oppression in the broader heteronormative culture are frequently mirrored in non-heterosexual online dating (e.g., Boyd Farmer and Byrd, 2015; Tang, 2017). The selected dating site also influences users by promoting a certain atmosphere through features such as “new face-pics” and “popular movies,” both mostly representing men and the latter promoting semi-nude men. In the end, the site operates within a structure that prioritizes profitability (Cavalcante, 2019) and features and ads that are profitable will remain. Discussions about self-presentations are important for current users as well as potential users. In coming-out processes, people often seek online communities where they acquire knowledge, language and norms of the LGBTQ community (Brown et al., 2005). The norms on this particular site are closely tied to the identified central themes.

### Strengths and Limitations

The present study’s believed strength lies in the novelty of the selected focus, examining a shared dating site for non-heterosexual men and women in a Nordic context. The combination of qualitative and quantitative findings from a rather large sample can contribute with knowledge to an under-researched topic and broaden the understanding of self-presentation on LGBTQ online dating sites in allegedly liberal countries. The study’s focus on gender on the mixed-gender site is considered a strength as previous research in large have focused on dating sites for gay men and/or limited gender analysis to ideals of masculinity and femininity amongst non-heterosexual men.

The key limitations in the present study are conceptual, as all concepts used are more or less subjective and open to alternative interpretations. The concepts used in the TA were generated from the data – not operationalized before analysis. Some of the selected concept, such as “body” are hopefully self-explanatory. Inherently subjective, the concepts were exemplified through selected quotes (exemplified definition). It every reader’s right to disagree with the choice of concepts. It is beyond the scope of the present study to linguistically and philosophically determine e.g., what “really” constitutes “self-presentation,” what “really” counts as “mind” etc. It should be noted that not all of the 716 online profiles examined in the present study had profile texts. The choice to not include any text in the profile, instead only self-presenting with a photo, and possible differences between those with and without profile texts are subjects for future studies. The qualitative findings are by nature subjective and contextual, and the author preserves openness to alternative interpretations (Willig, 2012). Additionally, different questions could have been asked about the data, generating different conclusions. It should be noted that the present study only represents cis-gendered and predominantly Swedish online dating profiles, and the findings are perhaps not easily generalizable beyond a Nordic, non-heterosexual context.

### A Sexualized Male Scene

The findings in the present study suggest that the self-presentations on the Nordic online dating scene both mimic and depart from gender-based norms in majority society. The Nordic countries’ self-image as gender-equal and LGBT progressive was not confirmed in the present study. The findings instead suggest that many men still perceive difficulties in being identified as non-heterosexual in the Nordic countries. The male self-sexualization combined with a general unwillingness to be identified, suggests that shamelessness could be operating as a concealer of shame. Avoiding disclosure of homosexuality can be done to avoid stigmatizing reactions (Schrimshaw et al., 2016), but it doesn’t really explain why this would be the case on the Nordic *LGBTQ* dating scene. It does not seem too farfetched to claim that non-heterosexual men on the selected site tend to self-present in a way that in the heterosexual world is more common amongst women, who in our patriarchal world still largely are evaluated in terms of their appearance (e.g., Jones, 2005; Agger, 2012). Among the men in the present study the self-objectification was often done in forceful way, self-presenting with e.g., erect genitalia and describing themselves as fuck-holes etc. Erections are public display of sought masculinity, and in displaying only genitalia, personality takes a backseat (Agger, 2012). By only presenting genitalia or a naked torso the user preserves anonymity, but at the same time appears to offer the body as a commodity separate from the rest of the person, assigned value equal to the sexual satisfaction it can supply to other (men) (Jones, 2005; Hall et al., 2012). For non-heterosexual women the opposite direction of self-presentations seems true, with a focus on the mind, on friendship and on dreams far from bodily desires and descriptions, in line with previous research about gender differences between non-heterosexual men and women (Hatala and Prehodka, 1996). Sex appears to disappear altogether among the women, in part possibly because many of them instead are pre-occupied with guarding their sexuality against male boundary breaking contact.

Gayness is still used as a “de-masculinizing” insult (Savenije, 2016) and at the root of the gay male idolization of masculinity, that marginalizes feminine/effeminate gay men, and lays sexism (Taywaditep, 2002; Clarkson, 2006; Eguchi, 2009; Murgo et al., 2017). Even if the self-objectification is tied to gender constructs, overt discussions about femininity and masculinity were not common in the present study, a fact that partially departs from previous research findings (e.g., Ward, 2008; Tziallas, 2015). This might in part be attributed to the presumed gender-equal Nordic setting (World Economic Forum, 2018), where women “officially” are portrayed as equal to men, which might cause a small spillover effect on the online dating scene.

As discussed in previous research (e.g., Ward, 2008; Tziallas, 2015) the highly body-oriented and sexualized self-presentations amongst non-heterosexual men can be viewed through several different lenses, from liberation and empowerment to degradation and self-violation. Open to alternative understandings, the present study’s stance is that the highly sexualized self-presentation among non-heterosexual men is something that at the very least should be a topic of inquiry. Problematizing sexual objectification and body ideals among non-heterosexual men is commonly perceived as a violation of the sexual liberation many gay men fought for, evoking fear that gay men’s sexual expression may become curtailed (Teunis, 2007). The present study believes that non-heterosexual men’s sexual expression is not the *only* expression that should be valued in the non-heterosexual online dating scene and that true equality can only exist when sexual minorities receive the same academic scrutiny as the majority society.

### Dating Misrepresentation

The LGBTQ community can be narrow-minded and discriminatory, where inclusion always leads to some form of exclusion (Valentine and Skelton, 2003; Casey, 2004; Brown, 2014). If *every* Nordic dating site for non-heterosexuals aims to cater to the sexual-oriented male profiler, inevitable other types of members risk feeling excluded. As could be seen in the present study, men who long for serious relationships and love loose visibility among the more sexualized profiles. This is unfortunate, as there exist several alternative sites (e.g., Grindr) for the more sexually inclined users, but few, if any, for the men who want to find a partner without being subjected to endless sexual photos and invites. Again, the Nordic LGBTQ scene is not huge and when one of the biggest online scenes, with institutional and cultural power, appears skewed in representation it raises questions. The present study’s intention is not to shame the individual self-objectifying man. Rather, the study wishes to highlight what seems to be a system failure, where the sexualized and sexualizing white male disproportionally dominates the Nordic LGBTQ online dating scene.

While it in most other Nordic settings would be considered highly problematic to highlight ethnicity as a variable of importance, the dating site departs from this by promoting ethnicity as one of its main search and self-identifying tools. The site’s anti-racism policies are contradicted by the promotion of racial inclusion and exclusion through the pre-programed “origin of look” variable, which increases the risk of objectification and discrimination (Finkel et al., 2012). In guarding boundaries, some profile texts unmistakably show that members on the site had been discriminated against on the basis of ethnicity and/or exotified and objectified. Most racist discrimination is, owing to the site’s policies, hidden in text messaging. Nearly 82% (182 women, 223 men) of the members who stated ethnicity, self-presented as North European, and there existed both a textual and a visual (subjective assessment) disproportionate lack of ethnic minorities on the site, pointing to a problematic misrepresentation. In line with previous research (Brennan et al., 2013; Boyd Farmer and Byrd, 2015), the white non-heterosexual man seems to be the most powerful within the selected dating site.

Discussing misrepresentation, the significance of gender on the Nordic LGBTQ online dating scene cannot be overestimated. Before the actual analysis departed, the first noticeable gender discrepancy was seen in the numbers of members, with three times as many men as women. There is no inherent logic to the numeric gender-imbalance. On the contrary, one could assume that there would be more women than men on the site as it is one of very few communities available for non-heterosexual women, while non-heterosexual men have several other online venues to connect on. There are generally few social spaces provided specifically for lesbians (Casey, 2004). In Sweden, one well-known site existed specifically for non-heterosexual women, but on its homepage “Sylvia.se” the following statement is all that remains: “Existed for girls who like girls, and their friends. Sylvia is disused. 2000 – 2019.” The reasons why dating sites for non-heterosexual women in general seem to have a lower success rate compared to sites for non-heterosexual men needs to be further addressed. That the online market for non-heterosexual women continues to be framed as a problem (Murray and Ankerson, 2016) could assumingly be attributed more to the lack of funding and profit, than to an unexplained disinterest among women to meet potential partners. The general scarcity of online spaces for Nordic non-heterosexual women makes it all the more important to point to the fact that the dominating mixed-gendered site genders its space increasingly male.

Men continually contacted women on the present LGBTQ site. This indicates male entitlement and reduces the chances of non-heterosexual women staying in the online LGBTQ dating community. In the end, it’s a question of oppression and (gender) misrepresentation. This is part of a broader problem, where women are excluded from different parts of the gay scene (Taylor, 2008; Ward, 2016). Queer women frequently feel undervalued, overlooked, and voiceless in LGBTQ communities (Boyd Farmer and Byrd, 2015). They also face risks of sexist backlash (Roth, 2016) and resistance from white gay men (Ward, 2016). If the men who persistently contacted women, who clearly stated a non-heterosexual orientation, are not (only) heterosexual men, assumingly bisexual men are also doing the boundary breaking on the present site. Non-heterosexual men have been found to match heterosexual men’s level of ignorance about feminism and can be just as sexist (Connell, 1992; Taywaditep, 2002; Zheng and Zheng, 2015; Lewis et al., 2018). Regardless of the sexual orientation of these men, it’s an issue of male entitlement and an obvious disregard of non-heterosexual women’s boundaries. Where and how non-heterosexual women in the Nordic countries will be able to meet potential partners in safe online spaces needs to be further addressed if non-heterosexual women’s dating opportunities truly are considered to be of equal importance as non-heterosexual men’s. According to the findings of the present study, the Nordic LGBTQ online is vastly dominated by white men. Further research and critical discussion on self-presentations, gender and possible discrimination on online dating landscapes for non-heterosexuals in rural areas, in small countries and on mixed gender-sites is advocated. One long-term goal of the present study is to implement the findings in a way that can help facilitate discussions and improvements concerning inclusion for minorities within minorities, be it non-heterosexual women, ethnic minorities, or other groups that are not sufficiently represented in the Nordic online dating scene of today.

## Data Availability Statement

The datasets generated for this study are available on request to the corresponding author.

## Ethics Statement

The study involving human participants was reviewed and approved by Etiknämnden i Umeå. Written informed consent was not required to participate in this study, in accordance with the national legislation and the institutional requirements.

## Author Contributions

EM came up with the idea for the study and completed it.

## Conflict of Interest

The author declares that the research was conducted in the absence of any commercial or financial relationships that could be construed as a potential conflict of interest.

## References

[B1] Agger B. (2012). *Oversharing – Presentations of Self in the Internet Age*, 2nd Edn New York, NY: Routledge.

[B2] Åkerlund M. (2019). Representations of trans people in Swedish newspapers. *Journal. Stud.* 20 1319–1338. 10.1080/1461670X.2018.1513816)

[B3] Attrill A. (2015). *The Manipulation of Online Self-Presentation – Create, Edit, Re-Edit and Present.* Hampshire: Palgrave Macmillan.

[B4] Bargh J. McKenna K. Fitzsimons G. (2002). Can you see the real me? Activation and expression of the “True Self” on the Internet. *J. Soc. Issues* 58 33–48. 10.1111/1540-4560.00247

[B5] Barrett L. (2009). The future of psychology: connecting mind to brain. *Perspect. Psychol. Sci.* 4 326–339. 10.1111/j.1745-6924.2009.01134.x 19844601 PMC2763392

[B6] Bell A. (2002). *Debates in Psychology (Routledge modular Psychology).* Hove: Psychology.

[B7] Björkenstam C. Andersson G. Dalman C. Cochran S. Kosidou K. (2016). Suicide in married couples in Sweden: is the risk greater in same-sex couples? *Eur. J. Epidemiol.* 31 685–690. 10.1007/s10654-016-0154-6 27168192 PMC6889060

[B8] Blackwell C. Birnholtz J. Abbott C. (2015). Seeing and being seen: co-situation and impression formation using Grindr, a location-aware gay dating app. *New Media Soc.* 17 1117–1136. 10.1177/1461444814521595

[B9] Boyd Farmer L. Byrd R. (2015). Genderism in the LGBTQQIA community: an interpretative phenomenological analysis. *J. LGBT Issues Couns.* 9 288–310. 10.1080/15538605.2015.110

[B10] Braun V. Clarke V. (2006). Using thematic analysis in psychology. *Qual. Res. Psychol.* 3 77–101. 10.1191/1478088706qp063oa

[B11] Brennan D. Asakura K. George C. Newman P. Giwa S. Hart T. (2013). “Never reflected anywhere”: body image among ethnoracialized gay and bisexual men. *Body Image* 10 389–398. 10.1016/j.bodyim.2013.03.006 23648108

[B12] Brown G. Maycock B. Burns S. (2005). Your picture is your bait: use and meaning of cyberspace among gay men. *J. Sex Res.* 42 63–73. 10.1080/00224490509552258 15795806

[B13] Brown M. (2014). Gender and sexuality II: there goes the gayborhood? *Prog. Hum. Geogr.* 38 457–465. 10.1177/0309132513484215

[B14] Buchanan E. A. Zimmer M. (2018). “Internet research ethics,” in *The Stanford Encyclopedia of Philosophy (Winter 2018 Edition)*, ed. Zalta E. N. (Stanford, CA: CSLI).

[B15] Callander D. Newman C. E. Holt M. (2015). Is sexual racism really racism? Distinguishing attitudes toward sexual racism and generic racism among gay and bisexual men. *Arch. Sex. Behav.* 44 1991–2000. 10.1007/s10508-015-0487-3 26149367

[B16] Carlson-Rainer E. (2017). Sweden is a world leader in peace, security, and human rights. *World Affairs* 180 79–85. 10.1177/0043820018759714

[B17] Casey M. (2004). De-dyking queer space(s): heterosexual female visibility in gay and lesbian spaces. *Sexualities* 7 446–461. 10.1177/1363460704047062

[B18] Cavalcante A. (2019). Tumbling into queer utopias and vortexes: experiences of LGBTQ social media users on Tumblr. *J. Homosex.* 66 1715–1735. 10.1080/00918369.2018.1511131 30235077

[B19] Choy C. H. (2018). Smartphone apps as cosituated closets: a lesbian app, public/private spaces, mobile intimacy, and collapsing contexts. *Mob. Media Commun.* 6 88–107. 10.1177/2050157917727803

[B20] Clarke V. Braun V. (2017). Thematic analysis. *J. Posit. Psychol.* 12 297–298. 10.1080/17439760.2016.1262613

[B21] Clarkson J. (2006). “Everyday Joe” versus “pissy, bitchy, queens”: gay masculinity on StraightActing.com. *J. Men’s Stud.* 14 191–207. 10.3149/jms.1402.191

[B22] Connell R. Messerschmidt J. (2005). Hegemonic masculinity: rethinking the concept. *Gender. Soc.* 19 829–859. 10.1177/0891243205278639)

[B23] Connell R. W. (1992). A very straight gay: masculinity, homosexual experience, and the dynamics of gender. *Am. Sociol. Rev.* 57 735–751.

[B24] Detournay D. (2019). The racial life of ‘Cisgender’: reflections on sex, gender and the body. *Parallax* 25 58–74. 10.1080/13534645.2019.1570606

[B25] Duguay S. (2019). ““There’s no one new around you”: Queer women’s experiences of scarcity in geospatial partner-seeking on Tinder,” in *The Geographies of Digital Sexuality”*, eds Nash C. J. Gorman-Murray A. (Singapore: Palgrave Macmillan).

[B26] Eguchi S. (2009). Negotiating hegemonic masculinity: the rhetorical strategy of “Straight-Acting” among gay men. *J. Intercult. Commun. Res.* 38 193–209. 10.1080/17475759.2009.508892

[B27] Ellison N. Hancock J. Toma C. (2012). Profile as promise: a framework for conceptualizing veracity in online dating self-presentations. *New Media Soc.* 14 45–62. 10.1177/1461444811410395

[B28] Ellison N. Heino R. Gibbs J. (2006). Managing impressions online:self-Presentation processes in the online dating environment. *J. Comput. Media. Commun.* 11 415–441. 10.1111/j.1083-6101.2006.00020.x

[B29] Eurostat, (2017). *Population Density, European Commission, Eurostat – Your key to European Statistics.* Available at: https://ec.europa.eu/eurostat/web/products-datasets/-/tps00003 (accessed January 3, 2019).

[B30] Farr D. (2011). Online women seeking women personal ads and the deployment of ‘Tomboy’ identities. *J. Lesbian Stud.* 15 493–506. 10.1080/10894160.2011.532035 21973069

[B31] Ferris L. Duguay S. (2019). “Tinder’s lesbian digital imaginary: investigating (im) permeable boundaries of sexual identity on a popular dating app,” in *New Media & Society*, ed. Jones S. (Thousand Oaks, CA: SAGE), 1–18. 10.1177/1461444819864903

[B32] Finkel E. J. Eastwick P. W. Karney B. R. Reis H. T. Sprecher S. (2012). Online dating: a critical analysis from the perspective of psychological science. *Psychol. Sci. Public Interest* 13 3–66. 10.1177/1529100612436522 26173279

[B33] Flick U. (ed.) (2014). *The SAGE Handbook of Qualitative Data Analysis.* Los Angeles, CA: SAGE.

[B34] Flores A. R. Park A. Lee Badgett M. V. (2018). *New Measures of LGBT Acceptance and Inclusion Worldwide.* Los Angeles, CA: The Williams Institute, UCLA School of Law.

[B35] Geist C. Reynolds M. Gaytán M. (2017). Unfinished business: disentangling sex, gender, and sexuality in sociological research on gender stratification. *Sociol. Comp.* 11:e12470 10.1111/soc4.12470

[B36] Goffman G. (1959/1990). *The Presentation of Self in Everyday Life.* London: Penguin Books.

[B37] Gosling J. (2018). Gender fluidity reflected in contemporary society. *Jung J.* 12 75–79. 10.1080/19342039.2018.14

[B38] Gudelunas D. (2012). There’s an app for that: the uses and gratifications of online social networks for gay men. *Sex. Cult.* 16 347–365. 10.1007/s12119-012-9127-4

[B39] Haavind H. Magnusson E. (2005). Feminism, psychology and identity transformations in the Nordic countries. *Femin. Psychol.* 15 236–247. 10.1177/0959353505051731

[B40] Hall P. C. West J. H. McIntyre E. (2012). Female self-sexualization in MySpace.com personal profile photographs. *Sex. Cult.* 16 1–16. 10.1007/s12119-011-9095-0

[B41] Hatala M. N. Prehodka J. (1996). Content analysis of gay male and lesbian personal advertisements. *Psychol. Rep.* 78 371–374. 10.2466/pr0.1996.78.2.371 9148289

[B42] Hewson C. Buchanan T. (eds) (2017). *Ethics Guidelines for Internet-Mediated Research. INF206/04.2017.* Leicester: British Psychological Society.

[B43] Hightower J. L. (2015). Producing desirable bodies: boundary work in a lesbian niche dating site. *Sexualities* 18 20–36. 10.1177/1363460714550900

[B44] Hobbs M. Owen S. Gerber L. (2017). Liquid love? Dating apps, sex, relationships and the digital transformation of intimacy. *J. Sociol.* 53 271–284. 10.1177/1440783316662718

[B45] Howitt D. (2016). *Introduction to Qualitative Research Methods in Psychology*, 3rd Edn Harlow: Pearson Education.

[B46] James D. Condie J. Lean G. (2019). “Travel, Tinder and gender in digitally mediated tourism encounters,” in *The Geographies of Digital Sexuality*, eds Nash C. J. Gorman-Murray A. (Singapore: Palgrave Macmillan).

[B47] Johansson K. Persson K. I. Deogan C. El-Khatib Z. (2018). Factors associated with condom use and HIV testing among young men who have sex with men: a cross-sectional survey in a random online sample in Sweden. *Sex. Trans. Infect.* 94 427–433. 10.1136/sextrans-2017-053369 29773663

[B48] Jones R. H. (2005). ‘You show me yours, I’ll show you mine’: the negotiation of shifts from textual to visual modes in computer-mediated interaction among gay men’. *Vis. Commun.* 4 69–92. 10.1177/1470357205048938

[B49] Kottorp A. Johansson K. Aase P. Rosenberg L. (2016). Housing for ageing LGBTQ people in Sweden: a descriptive study of needs, preferences, and concerns. *Scand. J. Occup. Ther.* 23 337–346. 10.3109/11038128.2015.1115547 26625160

[B50] Lagerberg R. (2018). *Working for a Gay-Friendly Sweden.* Available at: https://sweden.se/society/working-for-a-gay-and-equal-sweden/ (accessed January 7, 2019).

[B51] Lemke R. Weber M. (2017). That man behind the curtain: investigating the sexual online dating behavior of men who have sex with men but hide their same-sex sexual attraction in offline surroundings. *J. Homosex.* 64 1561–1582. 10.1080/00918369.2016.1249735 27754811

[B52] Lewis B. Hesse C. Cook B. Pedersen C. (2018). Sexistential crisis: an intersectional analysis of gender expression and sexual orientation in masculine overcompensation. *J. Homosex* 67 58–78. 10.1080/00918369.2018.1525943 30307840

[B53] Liinason M. (2011). The construction of gender research in Sweden: an analysis of a success story. *Queer Scope Art.* 5 30–43.

[B54] Linander I. Alm E. Goicolea I. Harryson L. (2019). “It was like I had to fit into a category”: Care-seekers’ experiences of gender regulation in the Swedish trans-specific healthcare. *Health* 23 21–38. 10.1177/1363459317708824 28523938

[B55] Lindqvist A. Renstrom E. A. Gustafsson Senden M. (2019). Reducing a male bias in language? Establishing the efficiency of three different gender-fair language strategies. *Sex Roles* 81 109–117. 10.1007/s11199-018-0974-9

[B56] Lundquist J. H. Lin K. (2015). Is love (color) blind? The economy of race among gay and straight daters. *Soc. Forc.* 93 1423–1449. 10.1093/sf/sov008

[B57] Magnusson E. (2011). Women, men, and all the other categories. *Nordic Psychol.* 63 88–114. 10.1027/1901-2276/a000034

[B58] Markham A. Buchanan E. (2012). *Ethical Decision-Making and Internet Research, Recommendations from the AoIR Ethics Working Committee (Version 2.0).* Available at: https://aoir.org/reports/ethics2.pdf (accessed September 3, 2018).

[B59] Mertens D. M. (2014). “Ethical use of qualitative data and findings,” in *The SAGE Handbook of Qualitative Data Analysis*, ed. Flick U. (Los Angeles, CA: SAGE).

[B60] Miles S. (2019). “Going the distance: locative dating technology and queer male practice-based identities,” in *The Geographies of Digital Sexuality*, eds Nash C. J. Gorman-Murray A. (Singapore: Palgrave Macmillan).

[B61] Miller B. (2015). “Dude, where’s your face?” Self-Presentation, self-description, and partner preferences on a social networking application for men who have sex with men: a content analysis. *Sex. Cult.* 19 637–658. 10.1007/s12119-015-9283-4

[B62] Murgo M. A. J. Huynh K. D. Lee D. L. Chrisler J. C. (2017). Anti-effeminacy moderates the relationship between masculinity and internalized heterosexism among gay men. *J. LGBT Issues Couns.* 11 106–118. 10.1080/15538605.2017.1310008

[B63] Murray S. Ankerson M. S. (2016). Lez takes time: designing lesbian contact in geosocial networking apps. *Crit. Stud. Media Commun.* 33 53–69. 10.1080/15295036.2015.113392

[B64] Nash C. (2013). The age of the “post-mo”? Toronto’s gay village and a new generation. *Geoforum* 49 243–252. 10.1016/j.geoforum.2012.11.023

[B65] Nash C. Gorman-Murray A. (2014). LGBT neighbourhoods and ‘new mobilities’: towards understanding transformations in sexual and gendered urban landscapes. *Int. J. Urb. Reg. Res.* 38 756–772. 10.1111/1468-2427.12104

[B66] Nash C. J. Gorman-Murray A. (2019). “Queer mobilities and new spatial media,” in *The Geographies of Digital Sexuality*, eds Nash C. J. Gorman-Murray A. (Singapore: Palgrave Macmillan).

[B67] Nordmarken S. (2019). Book Review: the remarkable rise of transgender rights by Jami K. Taylor, Daniel C. Lewis, and Donald P. Haider-Markel. *Gender Soc.* 33 667–669. 10.1177/0891243219837714

[B68] Parisi L. Comunello F. (2016). “Exploring networked interactions through the lens of location-based dating services – The case of Italian Grindr users,” in *LGBTQs, Media and Culture in Europe*, eds Dhoest A. Szulc L. Eeckhout B. (New York, NY: Routledge).

[B69] Peterson A. Wahlström M. Wennerhag M. (2018). ‘Normalized’ Pride? Pride parade participants in six European countries. *Sexualities* 21 1146–1169. 10.1177/1363460717715032

[B70] Phua V. C. Kaufman G. (2003). The crossroads of race and sexuality. *J. Fam. Issues* 24 981–994. 10.1177/0192513X03256607

[B71] Potârcã G. Mills M. Neberich W. (2015). Relationship preferences among gay and Lesbian online daters: individual and contextual influences. *J. Marriage Fam.* 77 523–541. 10.1111/jomf.12177

[B72] Rafalow M. H. Feliciano C. Robnett B. (2017). Racialized femininity and masculinity in the preferences of online same-sex daters. *Soc. Curr.* 4 306–321. 10.1177/232949651668662

[B73] Ranzini G. Lutz C. (2017). Love at first swipe? Explaining Tinder self-presentation and motives. *Mob. Media Commun.* 5 80–101. 10.1177/2050157916664559

[B74] Reynolds C. (2015). “I am super straight and I prefer you be too”: constructions of heterosexual masculinity in online personal ads for “straight” men seeking sex with men. *J. Commun. Inq.* 39 213–231. 10.1177/0196859915575736

[B75] Rich A. (1980). Compulsory heterosexuality and lesbian existence. *Signs* 5 631–660. 10.1086/493756

[B76] Riessman Kohler C. (2008). *Narrative Methods for the Human Sciences.* Los Angelses, CA: Sage Publications.

[B77] Robinson B. A. (2016). The quantifiable-body discourse: “Height-weight proportionality” and gay men’s bodies in cyberspace. *Soc. Curr.* 3 172–185. 10.1177/2329496515604638

[B78] Rosenfeld M. J. Byung-Soo K. (2005). Young adult relationship values at the intersection of gender and sexuality. *J. Marriage Fam.* 71 510–525. 10.1111/j.1741-3737.2009.00616.x 23710079 PMC3662253

[B79] Rosenfeld M. J. Reuben J. T. (2012). Searching for a mate: the rise of the internet as a social intermediary. *Am. Sociol. Rev.* 77 523–547. 10.1177/0003122412448050

[B80] Roth U. (2016). “Coming out in the digital age – The opportunities and limitations of internet use in queer-lesbian coming-out experiences in Germany,” in *LGBTQs, Media and Culture in Europe*, eds Dhoest A. Szulc L. Eeckhout B. (New York, NY: Routledge).

[B81] Savenije T. (2016). “Homosexuality on Dutch and Flemish Facebook pages,” in *LGBTQs, Media and Culture in Europe*, eds Dhoest A. Szulc L. Eeckhout B. (New York, NY: Routledge).

[B82] Schrimshaw E. W. Downing M. J. Cohn D. J. (2016). Reasons for non-disclosure of sexual orientation among behaviorally bisexual men: non-disclosure as stigma management. *Arch. Sex. Behav.* 47 219–233. 10.1007/s10508-016-0762-y 27278965 PMC5145776

[B83] Shield A. (2014). ‘Suriname - Seeking a lonely, lesbian friend for correspondence’: immigration and Homo-emancipation in the Netherlands, 1965-79. *Hist. Worksh. J.* 78 246–264. 10.1093/hwj/dbu020

[B84] Shield A. (2016). “New in town – Gay immigrants and geosocial media,” in *LGBTQs, Media and Culture in Europe*, eds Dhoest A. Szulc L. Eeckhout B. (New York, NY: Routledge).

[B85] Siverskog A. Bromseth J. (2019). Subcultural spaces: LGBTQ aging in a Swedish context. *Int. J. Aging Hum. Dev.* 88 325–340. 10.1177/0091415019836923 30913895

[B86] Smith A. Anderson M. (2015). *5 Facts About Online Dating.* Available at: http://www.pewresearch.org/fact-tank/2015/04/20/5-facts-about-online-dating/ (accessed September 14, 2018).

[B87] Stempfhuber M. Liegl M. (2016). Intimacy mobilized: hook-up practices in the location-based social network Grindr. *Österreichische Zeitschrift für Soziologie* 41 51–70. 10.1007/s11614-016-0189-7

[B88] Suler J. (2004). The online disinhibition effect. *Cyberpsychol. Behav.* 7 321–326. 10.1089/1094931041291295 15257832

[B89] Sundevall F. Persson A. (2016). LGBT in the military: policy development in Sweden 1944–2014. *Sex. Res. Soc. Policy* 13 119–129. 10.1007/s13178-015-0217-6 27195050 PMC4841839

[B90] Svensson J. (2015). Participation as a pastime: political discussion in a queer community online. *Javnost Public* 22 283–297. 10.1080/13183222.2015.1060014

[B91] Svensson J. (2016). “Gay the correct way – Mundane queer flaming practices in online discussions of politics,” in *LGBTQs, Media and Culture in Europe*, eds Dhoest A. Szulc L. Eeckhout B. (New York, NY: Routledge).

[B92] Tang D. T. (2017). All I get is an emoji: dating on lesbian mobile phone app Butterfly. *Media Cult. Soc.* 39 816–832. 10.1177/0163443717693680

[B93] Taylor Y. (2008). ‘That’s not really my scene’: working-class lesbians in (and out of) place. *Sexualities* 11 523–546. 10.1177/1363460708094266

[B94] Taywaditep K. (2002). Marginalization among the marginalized: Gay men’s anti-effeminacy attitudes. *J. Homosex.* 42 1–28. 10.1300/J082v42n01_01 11991561

[B95] Teunis N. (2007). Sexual objectification and the construction of whiteness in the gay male community. *Cult. Health Sex.* 9 263–275. 10.1080/13691050601035597 17457730

[B96] The International Lesbian, Gay, Bisexual, Trans, and Intersex Association, (2019). *Country Ranking – Rainbow Europe.* Available at: https://rainbow-europe.org/country-ranking (accessed January 3, 2019).

[B97] Timofejevs-Henriksson P. (2011). The (subtly) questioned love: a love exile in Sweden. *Signs* 36 806–811. 10.1086/658502

[B98] Tyler J. M. Feldman R. S. (2004). Truth, lies, and self-presentation: how gender and anticipated future interaction relate to deceptive behavior. *J. Appl. Soc. Psychol.* 34 12 10.1111/j.1559-1816.2004.tb01994.x

[B99] Tziallas E. (2015). Gamified eroticism: Gay male “social networking” applications and self-pornography. *Sex. Cult.* 19 759–775. 10.1007/s12119-015-9288-z

[B100] Valentine G. (2000). From nowhere to everywhere: Lesbian geographies. *J. Lesbian Stud.* 4 1–9. 10.1300/J155v04n01_0124802679

[B101] Valentine G. Skelton T. (2003). Finding oneself, losing oneself: the lesbian and gay ‘scene’ as a paradoxical space. *Int. J. Urb. Reg. Res.* 27 849–866. 10.1111/j.0309-1317.2003.00487.x

[B102] Ward J. (2008). Dude-sex: white masculinities and ‘authentic’ heterosexuality among dudes who have sex with dudes. *Sexualities* 11 414–434. 10.1177/1363460708091742

[B103] Ward J. (2016). Dyke methods: a meditation on queer studies and the gay men who hate it. *Women’s Stud. Q.* 44 68–85. 10.1353/wsq.2016.0036

[B104] Wilkinson W. (2008). Threatening the patriarchy: testing an explanatory paradigm of anti-lesbian attitudes. *Sex Roles* 59 512–520. 10.1007/s11199-008-9432-4

[B105] Willig C. (2012). *Qualitative Interpretation and Analysis in Psychology.* London: McGraw-Hill Education.

[B106] Willig C. (2013). *Introducing Qualitative Research in Psychology*, 3rd Edn London: Open University Press.

[B107] Wood M. (2004). The gay male gaze: body image disturbance and gender oppression among gay men. *J. Gay Lesbian Soc. Serv.* 17 43–62. 10.1300/J041v17n02_03

[B108] World Economic Forum, (2018). *The Global Gender Gap Report 2018.* Available at: http://www3.weforum.org/docs/WEF_GGGR_2018.pdf (accessed January 3, 2019).

[B109] Zhao S. G. S. Martin J. (2008). Identity construction on Facebook: digital empowerment in anchored relationships. *Comput. Hum. Behav.* 24 1816–1836. 10.1016/j.chb.2008.02.012

[B110] Zheng L. Zheng Y. (2015). Young gay men’s sexism predict their male facial masculinity preference in China. *Pers. Individ. Differ.* 76 183–186. 10.1016/j.paid.2014.12.022

